# Deep south-north genetic divergence in Godlewski’s bunting (*Emberiza godlewskii*) related to uplift of the Qinghai-Tibet Plateau and habitat preferences

**DOI:** 10.1186/s12862-019-1487-z

**Published:** 2019-08-01

**Authors:** Jiande Li, Gang Song, Naifa Liu, Yongbin Chang, Xinkang Bao

**Affiliations:** 1 School of Life Sciences, Lanzhou University, Lanzhou, 730000 China; 2 Key Laboratory of Zoological Systematics and Evolution, Institute of Zoology, Chinese Academy of Sciences, Beijing, 100101 China

**Keywords:** *Emberiza godlewskii*, Phylogeography, Uplift of the Qinghai-Tibet plateau, West Qinling Mountains, Historical demographic dynamics, Population expansion

## Abstract

**Background:**

Geological events and climatic changes played important roles in shaping population differentiation and distribution within species. In China, populations in many species have contracted and expanded responding to environmental changes with the uplift of the Qinghai-Tibet Plateau (QTP) and glacial cycles during Pleistocene. In this study, we analysed the population structure of Godlewski’s Bunting, *Emberiza godlewskii,* to determine the effects of major historical events, geographic barriers and past climatic changes on phylogenetic divergence and historical demographic dynamics of this species.

**Results:**

A phylogeny based on concatenated mitochondrial and nuclear DNA datasets show two (northern and southern) clades approximately diverged 3.26 million years ago (Ma). The West Qinling Mountains serve as a dividing line between the two lineages. Both lineages experienced a recent demographic expansion during interglacial periods (marine isotope stages (MISs) 2–6). Bayesian skyline plots and the results of ecological niche modelling suggested a more intensive expansion of the northern lineage during the late Pleistocene, whereas the southern lineage was comparatively mild in population growth.

**Conclusions:**

Our results provide insights into the distribution patterns of avian taxa and the possible mechanisms for a south and north divergence model in China. The deep divergence may have been shaped by the uplift of the QTP. Habitat preferences might have facilitated the lineage divergence for *E. godlewskii*. Moreover, the West Qinling Mountains act as a dividing line between the two lineages, indicating a novel phylogeographic pattern of organisms in China. The difference in population expansion mode between two lineages resulted from different effects caused by the climate of the LGM and the subsequent habitat changes accompanying the arrival of a colder climate in northern and southern regions of China.

**Electronic supplementary material:**

The online version of this article (10.1186/s12862-019-1487-z) contains supplementary material, which is available to authorized users.

## Background

The phylogeographic patterns of avian taxa in China are diverse [1]. Among them, the south-north divergence is not quite common for widely-distributed birds. For example, studies on generalist species such as the Black-billed Magpie, *Pica pica,* which inhabit various open and semi-open habitats including short-grass areas but depend on trees and tall bushes, did not find major divergence between northern and southern populations [2]. In contrast, the azure-winged magpie, *Cyanopica cyanus*, which requires well-wooded habitat, shows a north-south genetic division in eastern China [3]. Multiple factors contribute to the observed phylogeographic patterns of divergence in China, including environmental features [4], geographical barriers [5], glacial periods [6], habitat preferences of organisms [3]. It is important to combine geo-climatical and ecological components in interpreting phylogeographical patterns we observed from empirical studies.

Zoogeographical studies have shown that the Himalayan Mountains and the southwestern mountain systems in China are important avian biodiversity hotspots in the Northern Hemisphere [7] and the Qinling Mountains – Huai River has been regarded as an ecological and zoogeographical boundary between the Oriental and Palaearctic realms in China [8]. However, the roles of mountain system in southwest China are not substantially illustrated (but see literatures by Song et al. [9] and Qu et.al. [10]), and few study to demonstrated the barrier effects of Qinling Mountains-Huai River line on lineage diversification in birds but questioned by Song et al. [5]. Therefore, it is necessary to evaluate the phylogeographical impacts of these geographic features and their related geological events on birds in central and south China.

Old World buntings (genus *Emberiza*) are widely distributed across the Palearctic, from East Asia and the Himalayas to the Middle East and Africa [11]. The species occupy a variety of breeding habitats. Most prefer semi-open to open taiga forest habitats, steppes and forest edges to very dense forests [12, 13]. Godlewski’s Bunting, *Emberiza godlewskii*, shows distribution and habitat preferences typical of the genus. The species tends to select bushy and rocky hill slopes that are often near forests, thickets, ravines, and farm fields [12]. In China, Godlewski’s Bunting is mainly distributed in the foothills of the Tien Shan range and on the western rim of the Tarim Basin. The species is also distributed throughout Gansu and Qinghai Provinces, northern and western Sichuan Province, Tibet (mainly on the northern side of the Himalayan range), northern and western Yunnan Province, Guizhou and Guangxi Provinces, and the mountains from the north of Shanxi-Beijing-Hebei to the south of the Qinling range in China [12, 14]. Five [15, 16] or six subspecies [14] have been recognized according to geographical distribution and morphological characteristics. Cheng et al. [17] proposed the existence of a subspecies *styani* based on differences in morphology and allopatric distribution from other subspecies. In our research, we included six subspecies and considered *styani* have a special status.

Although numerous species diversified in the Old World, there are few studies on the phylogeography of *Emberiza.* Päckert et al. [11] studied the phylogeny of buntings and proposed that elevational segregation between alpine and lowland breeding habitats might be responsible for the northern and southern lineage separation between *E. g. yunnanensis* and its northern conspecifics. Nevertheless, due to limited geographic coverage and a small sample size, the genetic structure and population history of *E. godlewskii* is not clear. In addition, previous studies failed to provide information on detailed demographic changes during glacial-interglacial cycle and distribution shift of the species caused by suitable habitats change during the last interglacial (LIG) and Last Glacial Maximum (LGM).

In this study, we incorporate an intensive sampling coverage and integrative approaches combining phylogeography with an isolation-with-migration (IM) model [18] to study the range-wide phylogeographic pattern of *E. godlewskii*, and investigate demographic dynamics with hypothetical alternative scenarios. Our specific objectives are as follows: (1) To identify the factors driving the phylogeny of *E. godlewskii* across its distribution range, locate the divergence boundary and determine whether apparent gene flow and migration existed between the major lineages; (2) To reconstruct a detailed demographic history of the major lineages with approximate Bayesian computation (ABC) modeling [19] in order to evaluate the effects of Quaternary climatic changes, use ecological niche models (ENMs) [20] to explore whether genetic differentiation within regions occurred and whether the potential distribution of the bunting contracted into refugia during the LGM in response to suitable habitat changes during Pleistocene glacial-interglacial cycles, and examine the fragmentation (pre-Pleistocene ‘vicariance’ model) between the major populations in which genetic differentiation occurred according to a null model of isolation without migration.

## Methods

### Sample collection and laboratory work

**Fig. 1:**
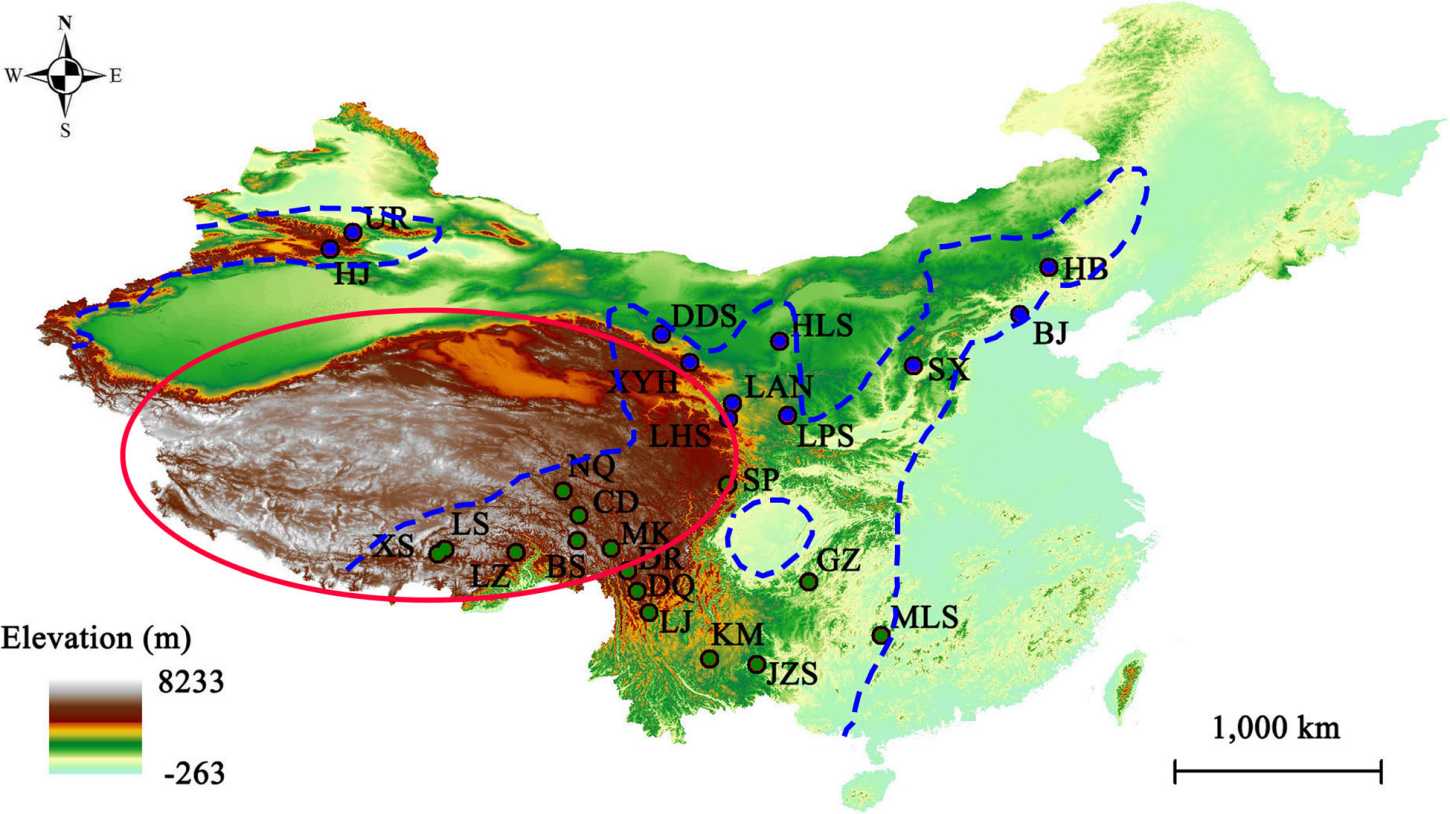
Sampling sites of *Emberiza godlewskii* in China (the map source is provided by Geospatial Data Cloud, Computer Network Information Center, Chinese Academy of Sciences (http://www.gscloud.cn)). The small circles represent our sampling sites, with blue indicating northern-lineage sites and green indicating southern-lineage sites. Capital letters denote the sampling localities s, which are presented in full in Additional file 1: Table S1. The area enclosed by blue dashed lines represents the distribution range of *E. godlewskii,* and the QTP is indicated with a red oval

We collected 190 blood samples from 26 sites covering the major distribution of Godlewski’s Bunting in China (see Fig. 1, Additional file 1: Table S1). Individuals were caught by mist net and blood samples were extracted from the vein under wing. All individuals were processed in the field and released immediately thereafter. The samples were preserved in anhydrous ethanol at − 20 °C and stored in the Zoological Laboratory of Lanzhou University.

Total genomic DNA was extracted using a TIANamp Genomic DNA Kit (Tiangen, Beijing, China). Cytochrome oxidase I (COI), cytochrome b (Cytb), the control region (CR) and two nuclear gene introns (beta-fibrinogen intron 5, FIB; muscle-specific kinase intron, MUSK) were amplified by polymerase chain reaction (PCR). The primer pairs and PCR conditions with optimized annealing temperatures are described in Additional file 1: Table S2. The PCR products were purified with a StarPrep Gel Extraction Kit and examined with 1% agarose gel electrophoresis. Sequencing was performed by the Sangon Biotech Company in Shanghai. The original sequences were assembled with SeqMan software (DNASTAR). The consensus sequences were aligned using ClustalW and manually proof read in MEGA.

### Genetic diversity and phylogenetic structure analyses

We calculated the genetic parameters of the population, including the number of segregating sites (S), the number of haplotypes (Nhap), haplotype diversity (Hd), and nucleotide diversity (Pi) in DnaSP 5.0. The heterozygous sequences of the two nuclear genes were phased using the program PHASE [21] with the default parameters.

We constructed the intraspecific phylogeny based on the concatenated mitochondrial haplotypes using a constant population size coalescent model in BEAST 1.8 [22]. The optimum model setting was determined by the results of MrModeltest 2.3 [23]. Because the sequence substitutions were expected to be constant at the intraspecific level, we applied the strict molecular clock model [24]. We ran 100 million generations for the mitochondrial DNA (mtDNA) data set of the Markov chain Monte Carlo (MCMC) chain, with a sampling frequency of 10000 generations. Convergence of the posterior distributions of the parameters was evaluated by monitoring the effective sample size (ESS > 200) and trace plots in Tracer1.5 [25]. A maximum-credibility tree, which represents the topology of maximum posterior probability, was calculated in TreeAnnotator 1.8 [22] after discarding the first 10% of trees as burn-in.

To test the validity of the major lineages revealed in the mtDNA, a species tree was estimated by *BEAST [26] based on mtDNA and nucler DNA (nuDNA) of 101 individuals. The *BEAST analysis was implemented in BEAST 2.0 [27]. We unlinked the substitution models across the five genes (Cytb, COI, CR, FIB and MUSK) and set the substitution parameters for each locus according to the MrModeltest results. We selected a piecewise linear and constant root model as the prior species tree and employed default molecular clock settings (strict clock model, rate of the first locus = 1.0, estimated rates of the remaining loci). The MCMC chains operated for 500 million generations. The sampling frequency was every 50000 generations, and the first 10% was discarded as burn-in. Convergence of the posterior distribution parameters was examined by monitoring the ESS (> 200) and trace plots in Tracer1.5 [25]. The results were visualized with DensiTree software [28]. We also reconstructed phylogenetic trees with MrBayes [29] and RAxML [30] based on concatenated fragments (mtDNA and nuDNA). The best-fitting model for the BI and RAxML analyses was determined separately for each gene at the five loci by MrModeltest 2.3 [23]. We included sequences of *Emberiza rutila* and *Emberiza leucocephalos* the outgroups. We conducted an analysis of molecular variance (AMOVA) based on FST to further determine where the major divergence occurred. We used AMOVA based on the concatenated mitochondrial set in Arlequin 3.5 [31] to diagnose population structure. As the sample sizes of some the populations, such as Lanzhou (LAN), Lianhuashan (LHS), Changdu (CD), Mangkang (MK), Deqin (DQ) and Derong (DR) were too small, we combined the LAN and LHS populations into LAN/LHS, the Hebei (HB) and Beijing (BJ) populations into HB/BJ, the CD and MK populations into CD/MK, the Lhasa (LS) and Xiongse (XS) populations into LS/XS, and the DQ and DR populations into DQ/DR according to the corrected average pairwise differences [pi XY- (pi X + pi Y)/2] and geographic conditions. The significance of each of the various components in the AMOVA was tested with 10000 permutations. Moreover, to verify the consistency of topological structure, we conducted clustering analyses of the median-joining networks with mtDNA to infer the intraspecific relationship among haplotypes using NETWORK 5.0 software [32].

### Divergence dating and historical demographic reconstruction

According to the phylogenetic results, we calculated the divergence time and reconstructed the demographic dynamics of the major lineages. Divergence times were estimated in BEAST 1.8 [22] based on the concatenated mitochondrial data set. We accepted the substitution rate of 0.01035 per site per million years (molecular clock for the passerine mitochondrial Cytb gene) [33] and modified it for the combined mtDNA sequences. We calculated the average overall mean p distances of Cytb and the combined mtDNA. The average overall mean p distances of Cytb and the combined mtDNA were 0.02100 and 0.02140, respectively, which implied a substitution rate of combined mtDNA 1.0190 times greater than that of Cytb. Therefore, we obtained a substitution rate of 0.01055 per site per million years for the combined mtDNA sequences [5]. The analyses were implemented with a constant growth as a coalescent constant tree prior and a strict molecular clock. The MCMC chains were run for 100 million generations with sampling every 10000 generations. We used Tracer 1.5 to examine the posterior distribution and ESSs and then summed the posterior probabilities of each parameter after discarding the first 10% of the samples as burn-in.

We calculated Tajima’s D [34], Fu’s Fs [35] and Ramos-Onsins’s and Rozas’s (R2) statistic [36] for each lineage to assess the neutral evolution of the mitochondrial genes. In Arlequin 3.5, we tested for historical demographic expansion using mismatch distribution analyses, and statistical significance was tested with two important parameters: the sum of squared deviations (SSD) and the raggedness index (r) between the observed and expected values [37]. The *P* values for these parameters were obtained from 1000 bootstrap samples.

To explore whether the major phylogenetic lineages experienced historical fluctuation, we conducted Bayesian skyline plots (BSPs) using mtDNA in BEAST 1.8 to reconstruct the changes in effective population size through time since the most recent common ancestor (TMRCA). We ran the analysis for 100 million generations or more until the ESSs were larger than 200, with sampling every 10000 generations after discarding the first 10% of samples as burn-in. Population curves of demographic history through time were reconstructed in Tracer 1.5.

To explore the demographic scenarios of divergence and expansion, we also applied approximate Bayesian computation (ABC) analysis in DIY-ABC, version 2.1.0. [38] with the mtDNA and nuDNA data sets. We adopted the divergence with isolation model based on the phylogenetic structure, which clearly displayed two lineages diverged from a common ancestor, and tested the possible scenarios of expansion for each lineage (Additional file 1: Figure S1). For the prior settings for parameters (Additional file 1: Table S3) in DIY-ABC, we used two groups: group 1, including 1.00E-9 to 1.00E-7 per site per year uniform priors, and group 2, including 1.00E-11 to 1.00E-8 per site per year uniform priors, for mtDNA and nuDNA respectively. We tested the following five scenarios of demographic changes for each lineage: a constant population size since divergence (scenario 1); recent expansion (scenario 2); old expansion (scenario 3); expansion-shrinkage (scenario 4); and expansion-shrinkage-expansion (scenario 5) [39] (Additional file 1: Figure S2a). To select the best model that could explain the genetic polymorphism, we simulated 1 000 000 multilocus genetic data sets for each scenario. The 1% of the simulated data sets closest to the observed data was used to estimate the relative posterior probability (with 95% confidence intervals (CIs)) of each scenario via logistic regression and posterior parameter distributions.

To better estimate gene flow and divergence time between the major lineages, we used IMa2 based on the IM model [40] with nuDNA and mtDNA data. In the analysis, demographic parameters, including divergence time (t), effective population size of each extant population (q0 and q1) and the ancestor (q2), and migration rates (m0 and m1) between two groups, scaled by the mutation rate (μ), were estimated. The maximum priors for the parameters used for IMa analyses were t = 0.48, q0 = q1 = q2 = 0.084, and m0 = m1 = 8.3. The inheritance scalars were set to 0.25 and 1 for mtDNA and nuDNA respectively. We performed multiple runs to ensure that posterior probability distributions converged by monitoring ESS values and trend lines along MCMC chains. In the simulations, we conducted each IMa simulation for 100000 steps with a burn-in of 100000 steps. The mtDNA and FIB loci were assumed to follow a Hasegawa-Kishino-Yano (HKY) model, and the MUSK locus was assumed to follow an infinite sites (IS) model of mutation, with a mean mutation rate value of 1.6E-8, 1.035E-8, 2.4E-8, 1.2E-9 and 1.9E-9 and confidence intervals of 1.0E-8 to 3.0E-8, 1.0E-8 to 3.0E-8, 1.0E-8 to 4.0E-8, 7.0E-10 to 2.0E-9 and 1.0E-9 to 3.0E-9 for COI, Cytb, CR, MUSK and FIB, respectively.

### Distribution changes with ecological niche modelling

To explore whether genetic differentiation within regions occurred in response to distribution changes of suitable habitat during Pleistocene glacial-interglacial cycles, we employed ENMs to predict the distribution of the *E. godlewskii* with 19 bio-climate variables from WorldClim [41] during four periods, namely, the present time (1960--1990 AD), the Mid-Holocene (MIH, 0.006 Ma), the LGM (0.021--0.018 Ma) and the LIG (0.14--0.12 Ma). We collected 766 occurrence records from the Global Biodiversity Information Facility (GBIF) (www.gbif.com), eBird, and our sampling sites, and after quality control steps to reduce spatial auto-correlation and sampling bias, we obtained 253 locations. We performed ENM analyses of the two different lineages based on the phylogenetic results, with 126 sites for the northern lineage and 127 sites for the southern lineage, with at least one of the longitude and latitude differences greater than 0.1° between every pair of locations. We constructed the model by randomly selecting 80% of the occurrence data, and the remaining 20% of the data were used to test the accuracy of the model in MaxEnt v3.3.3 k. The parameters were set to the default convergence threshold (10--5), 2000 maximum iterations and 10 replicates. MaxEnt was used to test the model by calculating the average area over ten replications under the receiver operating characteristic curve (AUC) and the binominal probabilities indicating the predictive ability of the model. Then, we reclassified the results into binary states under the 10% logistic threshold, indicating an unsuitable area, and higher than the threshold, indicating a suitable area.

## Results

### Genetic diversity and molecular evolution

We obtained 1103 base pairs (bp), 1323 bp, and 1094 bp of Cytb, COI and CR, respectively, for 190 individuals. For the nuclear gene introns, we obtained 509 bp and 558 bp of FIB and MUSK, repectively, for 101 individuals. All sequences were deposited in GenBank (see Additional file 1: Tables S4 and S5). The Cytb, COI, CR, and COI + Cytb+CR data sets contained 101, 103, 100 and 304 variable sites, defining 72, 75, 89 and 144 haplotypes, respectively. MUSK and FIB contained 16 and 62 variable sites, defining 18 and 70 haplotypes, respectively. CR had the highest Hd (0.968) and Pi (0.024) values among the mitochondrial genes, whereas FIB had larger Hd (0.960) and pi (0.018) values than MUSK. Neither Tajima’s D test nor Fu’s Fs indicated significant deviations from neutrality at the five loci, with the exception of a significantly negative Tajima’s D value (*P* < 0.05) for the MUSK gene (Table 1).

**Table 1:** Genetic diversity indices and results of neutrality tests for *Emberiza godlewskii*

	mtDNA				nuDNA	
	cytb	COI	CR	cytb+COI + CR	MUSK	FIB
Sample size	190	190	190	190	101	101
Length(bp)	1103	1323	1094	3520	558	509
S	101	103	100	304	16	62
Nhap	72	75	89	144	18	70
Hd	0.928	0.926	0.968	0.994	0.531	0.960
Pi	0.021	0.019	0.024	0.021	0.001	0.018
Tajima’s D	0.840	1.393	1.515	1.283	−1.841*	−0.482
Fu ‘s F	−1.683	− 0.177	−0.384	− 0.814	−0.539	− 1.035

*P < 0.05

*S* Number of segregating sites, *Nhap* Number of haplotypes, *Hd* Haplotype diversity, *Pi* Nucleotide diversity; *Fu’s F* Statistic of Fu and Li’s F* test, *Tajima’s D* Statistic of Tajima’s D test

### Intraspecific phylogeny and geographic divergence

**Fig. 2:**
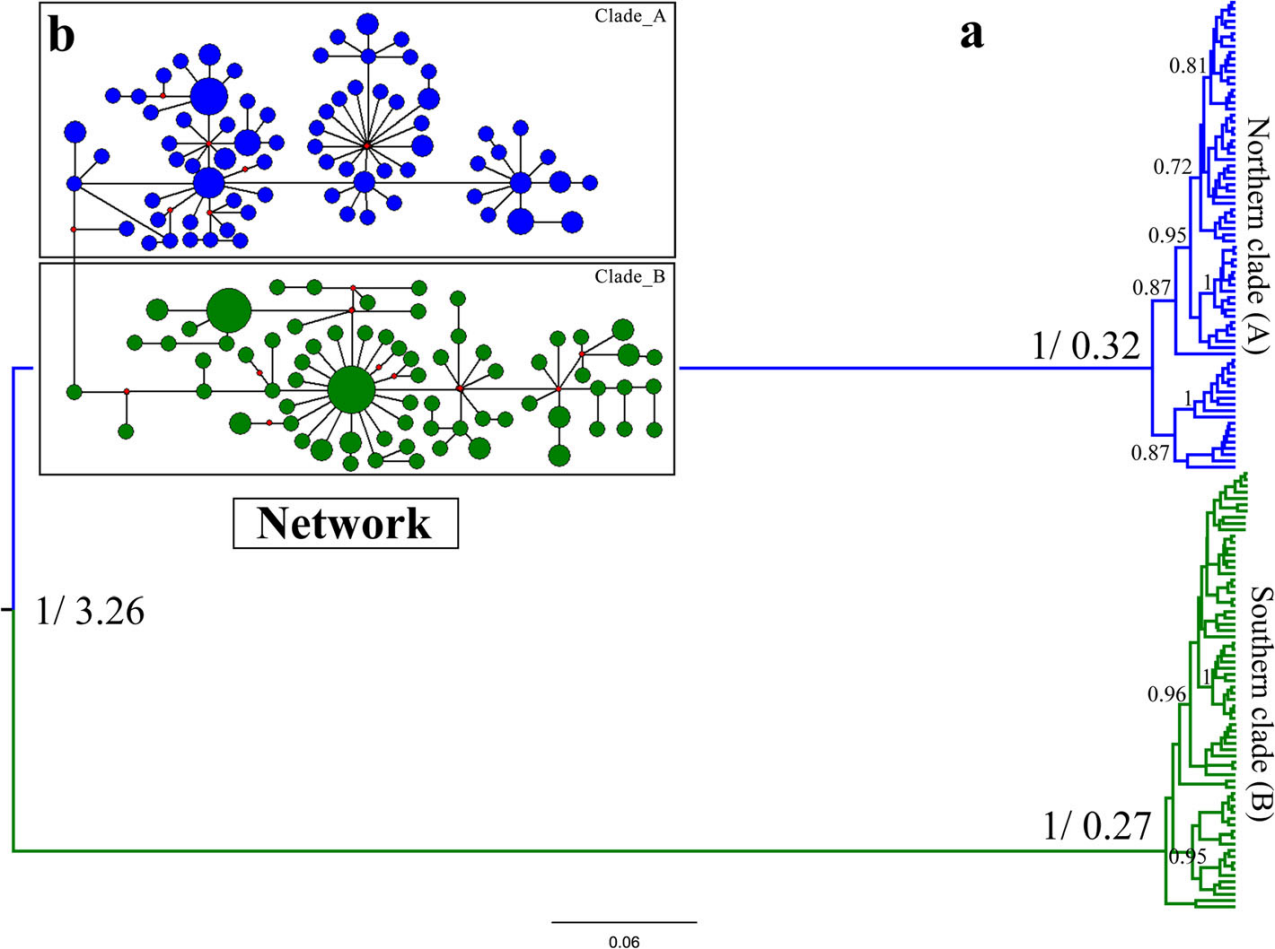
Phylogenetic relationships based on the haplotypes of concatenated mitochondrial genes (COI + Cytb+CR). **a** Bayesian trees. **b** Median-joining networks. The coloured bars indicate the geographical clades: blue denotes the northern clade (**a**) and green denotes the southern clade (**b**). The number on the left of a slash is the posterior probability and that on the right is divergence time. Posterior probabilities > 0.70 are labelled at each node. Red dots refer to missing steps intermediate between the observed haplotypes

**Fig. 3:**
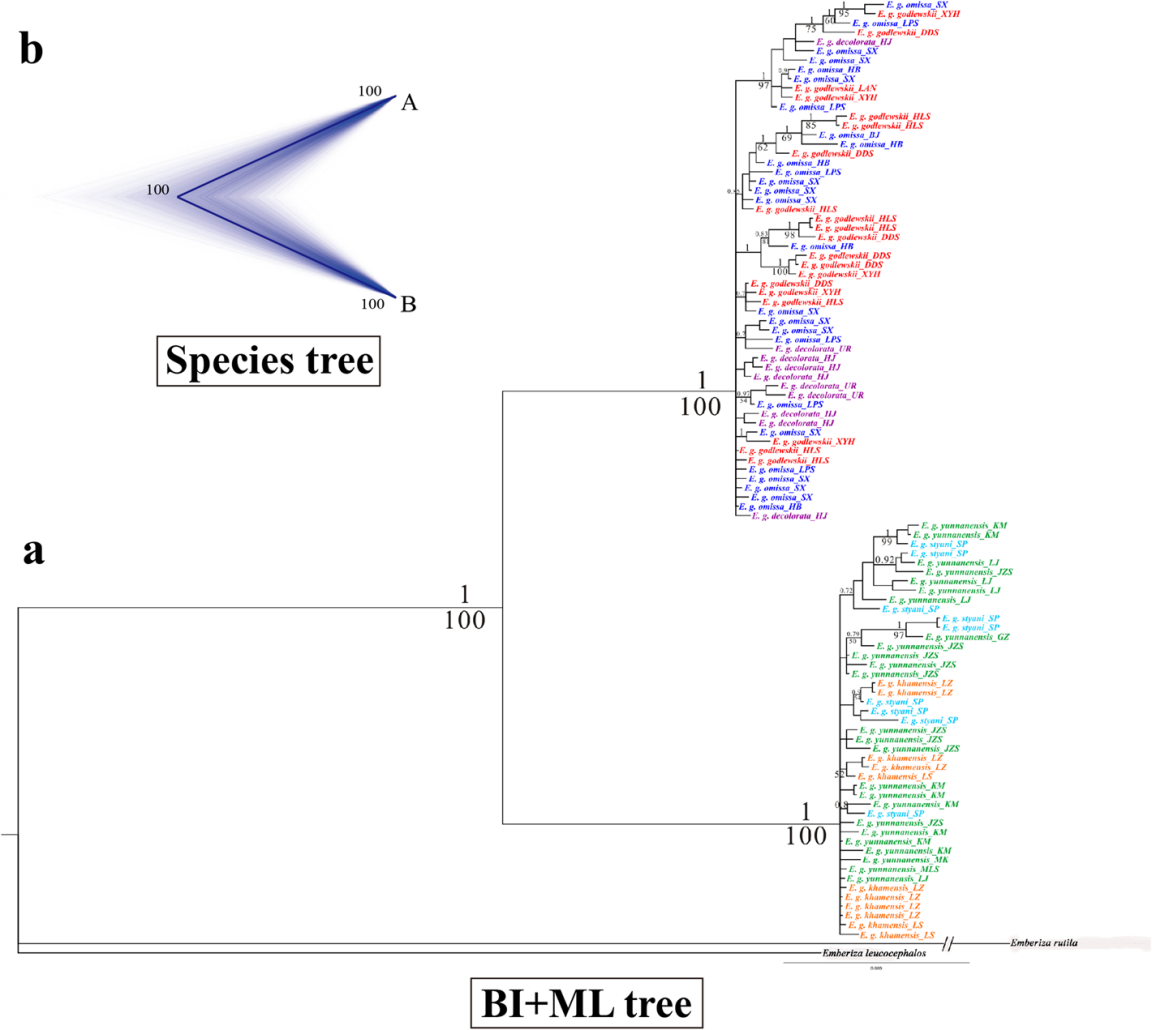
Phylogeny of individuals from China based on the mitochondrial and nuclear genes. **a** Phylogenetic trees were inferred by concatenation analyses, MrBayes and RAxML (BI+ML). **b** Species tree of major lineages estimated by *BEAST. The outgroup species were *Emberiza rutila* and *Emberiza leucocephalos*. Bootstraps values> 50 and posterior probabilities > 0.7 are labelled at each node. Different colours indicate different subspecies

The best molecular evolutionary model of the combined mtDNA was GTR + I + G. The best-fitting models of COI, Cytb, CR, MUSK and FIB were GTR + I, HKY + I, GTR + I + G, HKY and HKY + I, respectively. The intraspecific phylogeny based on mtDNA strongly supported deep divergence of the northern (clade A) and southern (clade B) clades in China (Fig. 2). There were no subclades corresponding to geographical subspecies. The RAxML, BI and *BEAST analyses recovered intraspecific phylogenies supported by high posterior probabilities and bootstrap values and that were consistent with the phylogenies from the mtDNA data (Fig. 3). The median-joining network of the mtDNA was topologically congruent with the trees (Figs. 2 and 3).

The results of the AMOVA based on the concatenated mtDNA showed that the maximum percentage (93.35%) of molecular variance resulted from between groups when the whole population localities were divided into northern and southern lineages. The variation percentages among the localities within group and within the populations were 0.89 and 5.75%, respectively (Table 2), and all of the *P* values were significant.

**Table 2:** AMOVA results based on mtDNA for *Emberiza godlewskii*

Grouping	Source of variation	d.f.	Percentage of variation
North/ South	Between groups	1	93.35
	Among localities within group	19	0.89
	Within populations	169	5.75

### Divergence dating and historical demographic reconstruction

The divergence time between clade A and clade B was dated to 3.26 (95% highest posterior density (HPD): 2.53–4.08) Ma, which was approximately in the late Pliocene (Table 3). The TMRCA of clade A was dated to 0.32 Ma (95% HPD: 0.21–0.38 Ma), and the TMRCA of clade B was dated to 0.27 Ma (95% HPD: 0.18–0.32 Ma). The divergence time estimated by IMa2 also showed divergence between the two lineages during approximately the late Pliocene. The maximum likelihood estimates of 2 Nm from north to south and vice versa were 0.091 and 0.077 migrants per generation, respectively (Table 4).

**Table 3:** Results of the Bayesian coalescent-based estimation of divergence time among different evolutionary clades and the time of the most recent common ancestor (TMRCA) of different evolutionary clades, 95% HPD values are shown in parentheses

Data sets	Molecular clock (site/million years)	Evolutionary clades	Divergence time estimate (Mya)
CO1 + cytb+CR	0.01055	cladeA vs cladeB	3.26 (2.53, 4.08)
		cladeA	0.32 (0.21, 0.38)
		cladeB	0.27 (0.18, 0.32)

**Table 4:** Maximum-likelihood estimates (MLE) and 90% highest posterior density (HPD) intervals of demographic parameters from IMA multilocus analyses

	t0	q0	q1	q2	2N0m0 > 1	2N1m1 > 0
MLEs	2.64E+ 06	1.66E+ 06	1.49E+ 06	1.60E+ 06	9.14E-02	7.68E-02
HPD95Lo	2.26E+ 06	8.10E+ 03	8.10E+ 03	2.11E+ 04	0.000	0.000
HPD95Hi	5.18E+ 07	3.24E+ 06	3.24E+ 06	3.08E+ 06	2.48E-01	2.65E-01

*t0* Time since major lineage divergence, *q0, q1, q2* Effective population size of northern population, southern population and ancestral population; *2N0m0 > 1* Population migration rate from the northern population to southern population migrants per generation; *2N1m1 > 0* Population migration rate from the southern population to northern population migrants per generation

**Fig. 4:**
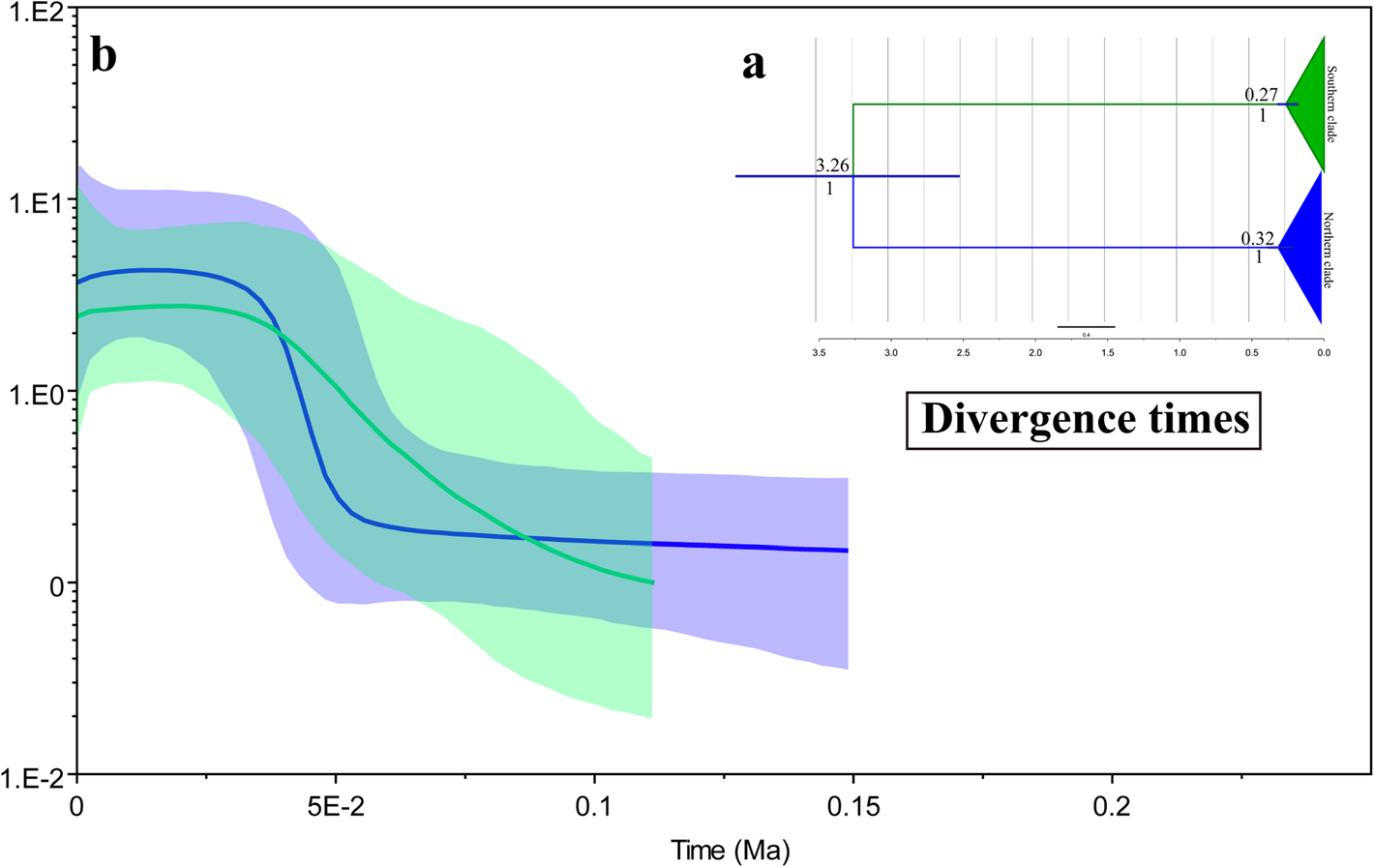
Calculated divergence times and historical demographic reconstruction based on mtDNA. **a** Estimation of the divergence time between lineages. The values below the node indicate Bayesian posterior probabilities, and values above the node represent divergence times. **b** BSP of the historical demographic trends of the two major lineages of *Emberiza godlewskii*. Blue denotes the northern lineage, and green denotes the southern lineage. The X axis is the timescale before present (Ma), and the Y axis is the estimated effective population size. Estimates of means are shown as solid lines, and the 95% HPDs are indicated by shaded areas

**Fig. 5:**
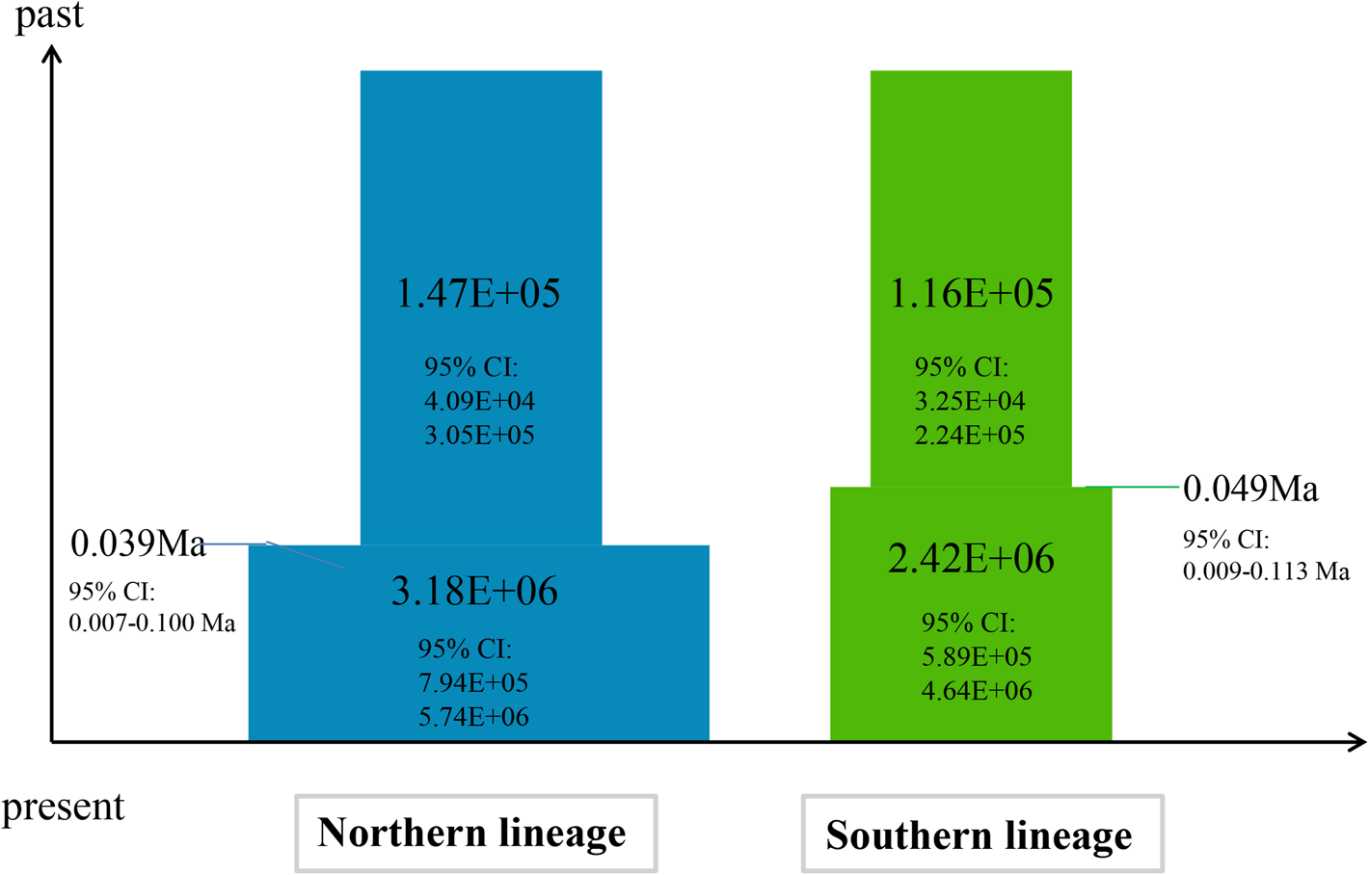
Summary of the inferred demographic history of the two genetic clades of *E. godlewskii*. Changes in population sizes are integrated into the divergence scenario. Mean population sizes with 95% CIs are indicated on each cylinder. Mean times of divergence with 95% CIs are indicated by fine horizontal lines

**Fig. 6:**
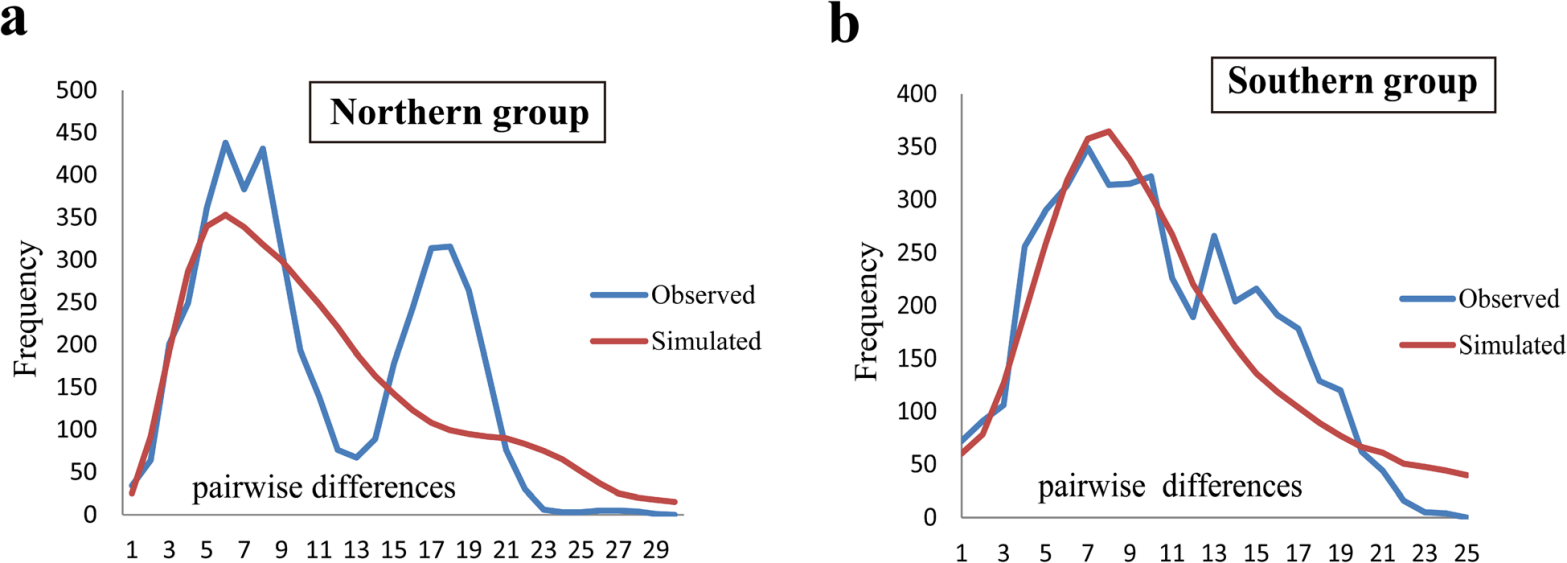
Mismatch distributions of the geographic groups of *Emberiza godlewskii* based on mtDNA. **a** The northern clade. **b** The southern clade. Each plot shows the frequency (Y axis) of pairwise nucleotide site differences (X axis) among sequences for each geographic group. The fit to the demographic expansion model is evaluated by the SSD and the r, which are shown in Table 4

The BSPs showed different demographic trends between the two lineages: The northern lineage showed rapid expansion, whereas the trend curve of the southern lineage was shallower (Fig. 4). Past population dynamics indicated that the rapid population growth of the northern lineage occurred 0.05 Ma whereas a mild expansion of the southern lineage occurred 0.12 Ma. The results of ABC (Table 5) for the divergence with isolation model indicated that the effective population sizes of the two groups were 6.97E+ 05 (95% CI: 4.15e+ 05 to 9.42e+ 05) and 6.23E+ 05 (95% CI: 3.41e+ 05 to 9.11e+ 05) and divergence time was 1.06E+ 06 (95% CI: 3.26e+ 05 to 4.42e+ 06) years ago. The logistic regression of the 1% of simulated data sets are similar to the observed data showed, the recent expansion (scenario 2) of both the northern and southern lineages fit best (Additional file 1: Figure S2b), indicating that both the northern and southern lineages experienced recent expansion (Fig. 5, Additional file 1: Table S6).Tajima’s D and Fu’s Fs values showed significant deviation from neutral evolution for both the northern (*P* values for Tajima’s D and Fu’s Fs: 0.001and 0.000, respectively) and southern clades (P values for Tajima’s D and Fu’s Fs: 0.003 and 0.000, respectively), and the values of R2 were small and positive (0.0309 and 0.0310, respectively) for both the northern and southern clades, indicating an excess of low-frequency mutations relative to expectations under the standard neutral model. The mismatch distributions of the northern (P values for the SSD and r: 0.430 and 0.370, respectively) and southern clades (P values for SSD and r: 0.740 and 0.880, respectively) fit the population expansion model, although the northern mismatch curve had two peaks. These results supported the model of historical demographic expansion (Table 6 and Fig. 6).

**Table 5:** Estimations of posterior distributions of parameters revealed by DIY-ABC for the changes in population sizes of the whole species population

Parameter	mean	median	mode	95%CI
N1	6.90e+ 005	6.97e+ 005	6.83e+ 005	4.15e+ 005 9.42e+ 005
N2	6.22e+ 005	6.23e+ 005	6.56e+ 005	3.41e+ 005 9.11e+ 005
t	1.51e+ 006	1.06e+ 006	6.37e+ 005	3.26e+ 005 4.42e+ 006
useq_1	2.66e-008	2.18e-008	1.81e-008	8.30e-009 6.27e-008
useq_2	5.82e-009	5.82e-009	6.11e-009	2.14e-009 9.47e-009

*N1* Effective population sizes of population 1, *N2* Effective population sizes of population 1, *t* Divergence time between population 1 and 2, *useq_1* The mutation rate of mtDNA, *useq_2* The mutation rate of nuDNA

**Table 6:** SSD and r indices of model fit for the model of demographic expansion. “Northern China” comprises the populations HJ, UR, DDS, XYH, LAN/LHS, LPS, HLS, SX and HB/BJ, and “Southern China” comprises the populations NQ, BS, LZ, CD/MK, LS/XS, SP, DQ/DR, GZ, JZS, KM, LJ and MLS

group	Tajmas’D	p	Fu’s Fs	p	SSD	p	r	p	R2
Northern China	−2.102	0.001	−24.498	0.000	0.010	0.430	0.006	0.370	0.0309
Southern China	−2.140	0.003	−24.592	0.000	0.002	0.740	0.003	0.880	0.0310

**Fig. 7:**
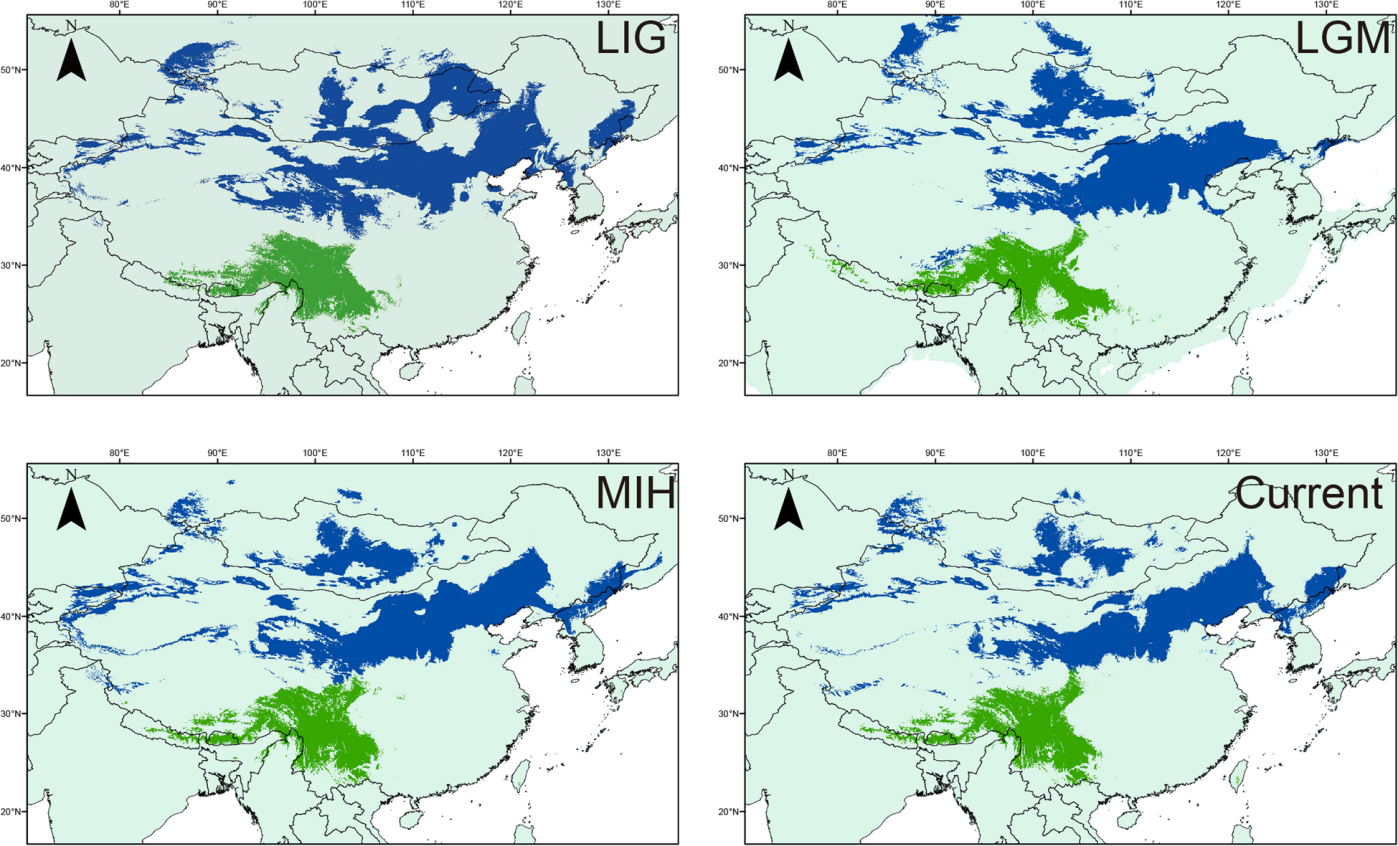
Predicted distributions of *E. godlewskii* based on ENMs using MaxEnt. Predicted distributions are shown for the LIG, LGM, MIH and CURRENT (the map source is cited from the Natural Earth (http://www.naturalearthdata.com/))

The ENM results (Fig. 7) showed that the LGM distribution was not consistent with the LIG distribution; in particular, the LGM distribution was different from the LIG and MIH/CURRENT distributions, suggesting that the distribution of the southern lineage experienced partial contraction and expansion during the LGM (25,000–15,000 years ago) and post-LGM, while the northern lineage contracted into North China from the LIG to the LGM and weakly expanded to the east and west after LGM.

## Discussion

### Deep north-south lineage divergence in *E. godlewskii*

The phylogenies showed that the *E. godlewskii* populations were divided into northern and southern clades. Each clade was further differentiated into subclades, but did not fully correspond to the subspecies. These findings indicate that subspecies of *E. godlewskii* cannot be genetically classified into simple groups and need re-evaluation.

As previously indicated by Päckert et al. [11], *E. godlewskii* was deeply divided into two lineages in China. The northern lineage was widely distributed in the northwest (Gansu, Xinjiang, Ningxia and Shaanxi) and north (Shanxi, Hebei and Beijing) of China, which are arid and semi-arid areas of the temperate zone. The southern lineage was distributed in the southeast of the Qinghai-Tibet Plateau (QTP) and the southwest (Yunnan, Guizhou, Guangxi and Sichuan) of China, corresponding to semi-humid areas of the subtropical zone. The belt in the narrow range from LHS to SP seems to act as the line dividing between the two lineages. The fragmentation of species distributions seems an important cause of allopatric divergence. Our ENM results indicated that there is a geographic boundary between the northern and southern clades. Moreover, IMa2 analysis confirmed a nearly null model of isolation without gene flow between the diverging populations, a gap between northern and southern populations. According to the divergence time estimated by the BEAST, the genetic differentiation might have been formed before the Pleistocene in a ‘vicariance’ model.

We propose that the deep divergence may be attributed to the uplift of the QTP, and a boundary corresponds to the West Qinling Orogenic Belt, located approximately 96°-106° E and 33°-37° N. The subduction collision and orogeny of this belt have been dated to the Silurian, but rapid uplift occurred in the late Pliocene [42]. Phylogenetic studies have revealed that some divergent events were consistent with the QTP uplift. Bao et al. [43] showed that the Tibetan partridge, *Perdix hodgsoniae*, diverged from its ancestor approximately 3.6 Ma, corresponding to the early intensive uplift of the QTP. The changes in climate, ecology, and habitats following the uplift of the QTP during the Pleistocene were likely the most important factors driving the pattern of phylogeography in Asian birds [1]. The difference in demographic dynamics and distribution range shifts between north and south lineages indicates that the specific habitat requirements of *E. godlewskii* was another important factor driving its expansion and shaping its distribution. The patterns of the south-north lineages are at least partly explained by ecological differences, especially in habitat preference.

The distribution pattern of *E. godlewskii* may be related to three geological events that occurred at different timescales in China. The first event was the early intensive uplift of the QTP, which may have been the major driver of the deep north-south split at 3.26 Ma, corresponding to the late Pliocene to early Pleistocene. The uplift of the QTP led to the geomorphological process of major downcutting in the Hengduan Mountain System [44], including the Yunnan-Guizhou Plateau [45], and shearing basins and graben valleys formed in the Southwest Mountains from the late Tertiary to the early Quaternary [46, 47]. These rugged basins or valleys at the edge of forests would have become refugia for *E. godlewskii* during glaciation. The second event consisted of ecological and climatic changes. During the uplift and the following climate fluctuations, the southeastern flanks of the Himalayan and Hengduan Mountains were part of the monsoon realm [48]. These areas were influenced by the southwestern monsoon winds that travelled deep into the continent through the north-south-oriented mountain ranges in Yunnan from the Indian Ocean [49]. Most of the areas were covered by subtropical mixed forest comprising evergreen broadleaf and broadleaf deciduous woodlands [1]. The dense forests separated the lowland and alpine-shrubland habitats at the forest edge into patches. At this time, in the intermontane basins of the Yunnan-Guizhou Plateau, the climate was dry and warm and the dominant vegetation in the late Pliocene was shrub meadow [50]. These conditions met the habitat requirements of *E. godlewskii.* The third event was the gradual shift to a drier and colder climate on the QTP, with the development of glaciers accompanying the uplift [51]. At this time, *E. godlewskii* was constricted into two lineages and dispersed to northern and southwestern China along both flanks of the western Qinling Mountains, which underwent extensive uplift along with the QTP and formed new basins for settlement [42]. The lineages refuged in the montane basins and gorges during the Quaternary glaciation. The species was separated into two lineages by the West Qinling Mountains, which is consistent with the traditional zoogeographical boundary between the Oriental and Palaearctic realms in China [52]. However, the observed phylogeographic pattern of the two lineages is at least partly explained by ecological dissimilarities within southern China (southern lineage) and northern China (northern lineage), especially in habitat preference.

### Divergence dating and historical demographic reconstruction

Another important question need to be addressed is whether apparent gene flow corresponding to secondary contact and admixture between the two lineages exits in a continuous time period. However, the results from IMa2 indicated no significant historical gene flow between the southern and northern lineages. Therefore, we simulated the demographic expansion of major lineages of this species based on a null model of isolation without migration (gene flow) between diverging populations with DIY-ABC, and the results indicated that both lineages underwent recent demographic expansions. The BSP indicated that the population experienced an expansion earlier than the LGM, predominantly during the interglacial periods (MISs 2–6) [1]. The northern and southern lineages expanded 0.05 Ma and 0.12 Ma, respectively, during the last interglacial period [53]. The demographic history analysis indicated that the distribution of this bird expanded before the LGM. This time is in contrast to the post-LGM expansion observed in most European bird populations and reflects the different timescales and frequencies of glaciations that occurred in Europe and Asia during the Pleistocene [6]. In fact, a warmer climate in the LIG provided opportunities for each lineage to expand their distribution range. However, the ENM showed different modes of distribution range shift in the two lineages, which may have resulted from different effects caused by the climate of the LGM and the subsequent habitat changes accompanying the arrival of a colder climate in northern and southern regions in China. For the southern lineage, the LGM distribution was contracted compared to the LIG, MIH and CURRENT. This likely relate to the landform heterogeneity in the Southwest Mountains of China [1, 54]. This coherent with BSP result of a mild expansion in the southern lineage. While for the northern lineage, a convergence was occurred and which contracted together into some refugia during the LGM, followed by partial expansion in the MIH after the LGM. This pattern may be related to the relative homogeneity of landforms in northern China, where the bird populations were more likely to be affected by the colder climate in the LGM.

The diverse local-topography and heterogonous climates in China during the Pleistocene can be expected to have varied effects on species-distributions and historical demography. As revealed by Su et al. [55], the climate during Pleistocene displayed an overall warm and humid trend, with a series of warming and cooling cycles. The vegetation in the Jiuquan Basin included spruces, pine, sagebrush and Solanaceae species approximately 0.04–0.012 Ma. Shi et al. [53] described a warm stage during the interstage of the last glaciation (0.06–0.03 Ma), when the temperature was approximately 3–4 °C higher than it is today. In the MIS3a stage (0.035 Ma), when the temperature in the Tsaidam Basin was 2 °C higher than today, the alpine and sub-alpine meadows turned into alpine coniferous forests and meadows in Zoige [56], and the distribution of vegetation expanded towards northern and western China [57]. These findings indicate that the forest developed and shrub meadow habitats arose near forest edges in northwestern China. Therefore, *E. godlewskii* rapidly expanded along with the suitable habitat in northern China in a short period of time, as indicated by the BSP. However, the glaciers in southwestern China retreated during MIS5 (0.11–0.071 Ma) [58], and a series of prolonged, mild interglacial periods developed [59], corresponding to the unique warming stage, MIS5e (0.125 Ma), in the LIG period [56]. Pollen studies have indicated that both alpine meadow and steppe had spread to the eastern part of the plateau during the glaciations [60]. Therefore, it is possible that this species expanded its distribution range during the warmer MIS5 period of the mild interglacial period, which resulted in vegetation similar to the present-day flora of East Asia [59].

## Conclusions

With a detailed phylogeographic structure of Godlewski’s Bunting, *E. godlewskii*, we provide new insights into the potential mechanisms of genetic divergence and historical dynamics of avian taxa in China. A south-north lineage divergence in *E. godlewskii* was identified and inferred related to geographic barrier of the West Qinling Mountains. The observed phylogeographic pattern may also have been driven by series geological events like uplift of QTP and subsequent environmental changes in glaciation. In particular, the two lineages showed different models of expansion in response to habitat changes during the Pleistocene glacial-interglacial cycles. Our findings may inform future investigations and potential taxonomic revisions.

## Additional file

Additional file 1: **Table S1** The sampling localities for Emberiza godlewskii. **Table S2** The PCR primers used in this study. **Table S3** Descriptions of prior settings for all parameters used in DIY-ABC. **Table S4** Genbank Accession numbers for individuals of mitochondrial genes used in this study. **Table S5** Genbank Accession numbers for individuals of nuclear genes used in this study. **Table S6** Estimations of posterior distributions of parameters revealed by DIY-ABC for the best scenario of demographic history of northern and southern population, respectively. **Figure S1** Demographic expansion and divergence models of Emberiza godlewskii. ABC results showing demographic history. Effective population sizes are marked in different colours and times of events (not to scale) are indicated. **Figure S2** (a) Schematic representation of ABC modelling of changes in population sizes. Na, ancestral population size; N1, current population size; Ne and Nb, population sizes between Na and N1; t2, old expansion time; tb, bottleneck time; t1, recent expansion time. (b) Posterior probabilities obtained by logistic regression of 1% of the closest simulated datasets for the northern and southern lineages, respectively, from left to right. (DOCX 279 kb)

## Data Availability

All sequences of Godlewski’s Bunting generated during the current study are available in the NCBI GenBank database under accession numbers MG194539-MG194728 and MG228461-MG229050. The data sets supporting the results and conclusions of this article are available in Additional file 1: Tables S3 and S4.

## Abbreviations

AMOVA: Analysis of molecular variance
BI: Bayesian inference
BJ: Beijing
BS: Basu
BSP: Bayesian skyline plots
CD: Changdu
COI: Cytochrome oxidase I
CR: Control region
Cytb: Cytochrome b
DDS: Dongda Mountain
DNA: Deoxyribonucleic acid
DQ: Deqin
DR: Derong
ESS: Effective sample size
FIB: Beta-fibrinogen intron 5
GZ: Guizhou
HB: Hebei
Hd: Haplotype diversity
HJ: Hejing
HLS: Helan Mountains
JZS: Jinzhong Mountain
KM: Kunming
LAN: Lanzhou
LGM: Last Glacial Maximum
LHS: Lianhuashan
LJ: Lijiang
LPS: Liupan Mountain
LS: Lhasa
LZ: Linzhi
Ma: Million years ago
MCMC: Markov chain Monte Carlo
MISs: Marine isotope stages
MK: Mangkang
ML: Maximum likelihood
MLS: Mao’er Mountain
mtDNA: Mitochondrial DNA
MUSK: Muscle-specific kinase intron
Nhap: Number of haplotypes
NQ: Nangqian
PCR: Polymerase chain reaction
pi: Nucleotide diversity
QTP: Qinghai-Tibet Plateau
r: Raggedness index
R2: Ramos-Onsins and Rozas
S: Number of segregating sites
SP: Songpan
SSD: Sum of squared deviations
SX: Shanxi
TMRCA: Time of the most recent common ancestor
UR: Urumchi
XS: Xiongse
XYH: Xiyinghe
