# Supplementary material for: Deep south-north genetic divergence in Godlewski’s bunting (Emberiza godlewskii) related to uplift of the Qinghai-Tibet Plateau and habitat preferences
Source: BMC Evol Biol. 2019 Aug 1;19:161. doi: 10.1186/s12862-019-1487-z (PMC6676563; doi:10.1186/s12862-019-1487-z)
Supplement: Supplementary file 1 — Table S1 The sampling localities for Emberiza godlewskii. Table S2 The PCR primers used in this study. Table S3 Descriptions of prior settings for all parameters used in DIY-ABC. Table S4 Genbank Accession numbers for individuals of mitochondrial genes used in this study. Table S5 Genbank Accession numbers for individuals of nuclear genes used in this study. Table S6 Estimations of posterior distributions of parameters revealed by DIY-ABC for the best scenario of demographic history of northern and southern population, respectively. Figure S1 Demographic expansion and divergence models of Emberiza godlewskii. ABC results showing demographic history. Effective population sizes are marked in different colours and times of events (not to scale) are indicated. Figure S2 (a) Schematic representation of ABC modelling of changes in population sizes. Na, ancestral population size; N1, current population size; Ne and Nb, population sizes between Na and N1; t2, old expansion time; tb, bottleneck time; t1, recent expansion time. (b) Posterior probabilities obtained by logistic regression of 1% of the closest simulated datasets for the northern and southern lineages, respectively, from left to right. (DOCX 279 kb) [file 12862_2019_1487_MOESM1_ESM.docx]

**Table S1.** The sampling localities for *Emberiza godlewskii.*

| Sampling locations | Exact location | Province | Sample size | Latitude Longitude | Altitude (m) |
| --- | --- | --- | --- | --- | --- |
| HJ | Hejing | Xinjiang | 7 | 42.4515˚N 86.1901 ˚E | 1102 |
| UR | Urumchi | Xinjiang | 5 | 43.2900˚N 87.1821˚E | 834 |
| DDS | Dongda mountain | Gansu | 9 | 39.0307˚N 100.4621˚E | 1906 |
| LAN | Lanzhou | Gansu | 2 | 36.0120˚N 103.5222˚E | 1536 |
| LHS | Lianhua mountain | Gansu | 2 | 35.3637˚N 103.7144˚E | 2449 |
| HLS | Helan mountain | Ningxia | 13 | 38.4422˚N 105.5633 ˚E | 1157 |
| XYH | Xiyinghe | Gansu | 10 | 37.4810˚N 102.0016 ˚E | 1523 |
| BJ | Beijing | Beijing | 2 | 39.9042˚N 116.4073 ˚E | 54 |
| HB | Hebei | Hebei | 13 | 41.5729˚N 117.4141 ˚E | 1120 |
| LPS | Liupan mountain | Ningxia | 8 | 35.2955˚N 106.1655 ˚E | 2253 |
| SX | Shanxi | Shanxi | 26 | 37.4038˚N 111.4651 ˚E | 1772 |
| NQ | Nangqian | Qinghai | 7 | 31.5929˚N 96.3046 ˚E | 3656 |
| BS | Basu | Xizang | 4 | 30.0133˚N 97.0626 ˚E | 3268 |
| CD | Changdu | Xizang | 1 | 31.1458˚N 97.1836 ˚E | 3296 |
| MK | Mangkang | Xizang | 2 | 29.6151˚N 98.6143 ˚E | 3874 |
| LS | Lhasa | Xizang | 4 | 29.3959˚N 91.1926 ˚E | 3653 |
| XS | Xiongse | Xizang | 4 | 29.2634˚N 91.0130 ˚E | 4086 |
| LZ | Linzhi | Xizang | 8 | 29.3127˚N 94.2555 ˚E | 3005 |
| SP | Songpan | Sichuan | 17 | 32.2846˚N 103.3932 ˚E | 3260 |
| DQ | Deqing | Yunnan | 1 | 27.8187˚N 99.7022 ˚E | 3217 |
| DR | Derong | Sichuan | 2 | 28.7130˚N 99.2863 ˚E | 2959 |
| GZ | Guizhou | Guizhou | 3 | 28.1356˚N 107.1252 ˚E | 1317 |
| JZS | Jinzhong mountain | Guangxi | 11 | 24.3806˚N 104.5715 ˚E | 287 |
| KM | Kunming | Yunnan | 13 | 24.5149˚N 102.5159 ˚E | 1894 |
| LJ | Lijiang | Yunnan | 13 | 26.5457˚N 100.1355 ˚E | 2377 |
| MLS | Mao’er mountain | Guangxi | 3 | 25.5322˚N 110.2242 ˚E | 224 |

**Table S2**. The PCR primers used in this study

| Locus | Name | Sequence(5’–3’) | T_Annealing_ (℃) | References |
| --- | --- | --- | --- | --- |
| cytb | L15353  H16065b | GTAATTACAAACCTATTCTCAGC  TTGGCTTACAAGACCAATGTTTT | 48 | [Kimball *et al.*, 2009](#_ENREF_37) |
| COI | L6615  H7956 | CCTATATAAAAAGGTCTACAGCC  GGGTAGTCGGAGTATCGACG | 51 | [Kimball *et al.*, 2009](#_ENREF_37) |
| CR | L15526  H16414 | CCTACGACCTGAAAAGCC  TGAATGGGGGTCAAAGTGC | 49 | [Kimball *et al.*, 2009](#_ENREF_37) |
| FIB | Fib5  Fib6 | CGCCATACAGAGTATACTGTGACAT  GCCATCCTGGCGATTCTGAA | 54 | [Kimball *et al.*, 2009](#_ENREF_37) |
| MUSK | MUSK-I3F  MUSK-I3R | CTTCCATGCACTACAATGGGAAA  CTCTGAACATTGTGGATCCTCAA | 50 | [Kimball *et al.*, 2009](#_ENREF_37) |

**Table S3. Descriptions of prior settings for all parameters used in DIY-ABC**

| Parameters | Distributions | |
| --- | --- | --- |
|  | Northern lineage | Southern lineage |
| N1 | Uniform[1E4-6E6] | Uniform[1E4-5E6] |
| t1 | Uniform[1E1-6E4] | Uniform[1E1-8E4] |
| Na | Uniform[1E4-3.5E5] | Uniform[1E4-2.5E5] |
| t2 | Uniform[1E1-1.2E6] | Uniform[1E1-2.2E6] |
| Ne | Uniform[1E4-1.4E7] | Uniform[1E4-1.8E7] |
| Nb | Uniform[1E4-3.5E5] | Uniform[1E4-2.5E5] |
| tb | Uniform[1E1-5E5] | Uniform[1E1-1E6] |
| ALL |  |  |
| N1 | Uniform[1E4-1E6] |  |
| N2 | Uniform[1E4-1E6] |  |
| t | Uniform[1E-1E6] |  |

(Fig. S2a) – examination of five demographic models of change in population size in two genetic groups, respectively. Population size parameters are in units of population effective size, while time parameters are in units of generations.

Prior settings for two scenarios northern and southern groups: Na, ancestral effective population size; N1, Effective population size after recent expansion; Nb, Effective population size during bottleneck; Ne, Effective population size after ancestral expansion; t2, ancestral population expansion time; tb, bottleneck time; t1, recent population expansion time.

Prior settings for scenario ALL: the effective population sizes of the whole species population; N1: effective population size of population 1 since divergence; N2: effective population size of population 2 since divergence; t1: the time of introduced events translated into two generations.

**Table S4**. Genbank Accession numbers for individuals of mitochondrial genes used in this study.

| Individual ID | Genbank Accession Number | | |
| --- | --- | --- | --- |
|  | *COI* | *Cytb* | *D-loop* |
| E_g_decolorata_HJ1 | MG194539 | MG228461 | MG228651 |
| E_g_decolorata_HJ2 | MG194540 | MG228462 | MG228652 |
| E_g_decolorata_HJ3 | MG194541 | MG228463 | MG228653 |
| E_g_decolorata_HJ4 | MG194542 | MG228464 | MG228654 |
| E_g_decolorata_HJ5 | MG194543 | MG228465 | MG228655 |
| E_g_decolorata_HJ6 | MG194544 | MG228466 | MG228656 |
| E_g_decolorata_HJ7 | MG194545 | MG228467 | MG228657 |
| E_g_decolorata_UR1 | MG194546 | MG228468 | MG228658 |
| E_g_decolorata_UR2 | MG194547 | MG228469 | MG228659 |
| E_g_decolorata_UR3 | MG194548 | MG228470 | MG228660 |
| E_g_decolorata_UR4 | MG194549 | MG228471 | MG228661 |
| E_g_decolorata_UR5 | MG194550 | MG228472 | MG228662 |
| E_g_godlewskii_DDS1 | MG194551 | MG228473 | MG228663 |
| E_g_godlewskii_DDS10 | MG194552 | MG228474 | MG228664 |
| E_g_godlewskii_DDS11 | MG194553 | MG228475 | MG228665 |
| E_g_godlewskii_DDS2 | MG194554 | MG228476 | MG228666 |
| E_g_godlewskii_DDS3 | MG194555 | MG228477 | MG228667 |
| E_g_godlewskii_DDS4 | MG194556 | MG228478 | MG228668 |
| E_g_godlewskii_DDS6 | MG194557 | MG228479 | MG228669 |
| E_g_godlewskii_DDS7 | MG194558 | MG228480 | MG228670 |
| E_g_godlewskii_DDS9 | MG194559 | MG228481 | MG228671 |
| E_g_godlewskii_LAN1 | MG194560 | MG228482 | MG228672 |
| E_g_godlewskii_LAN2 | MG194561 | MG228483 | MG228673 |
| E_g_godlewskii_LHS2 | MG194562 | MG228484 | MG228674 |
| E_g_godlewskii_LHS3 | MG194563 | MG228485 | MG228675 |
| E_g_godlewskii_NQ1 | MG194564 | MG228486 | MG228676 |
| E_g_godlewskii_NQ2 | MG194565 | MG228487 | MG228677 |
| E_g_godlewskii_NQ3 | MG194566 | MG228488 | MG228678 |
| E_g_godlewskii_NQ4 | MG194567 | MG228489 | MG228679 |
| E_g_godlewskii_NQ5 | MG194568 | MG228490 | MG228680 |
| E_g_godlewskii_NQ6 | MG194569 | MG228491 | MG228681 |
| E_g_godlewskii_NQ7 | MG194570 | MG228492 | MG228682 |
| E_g_godlewskii_SY1 | MG194571 | MG228493 | MG228683 |
| E_g_godlewskii_SY10 | MG194572 | MG228494 | MG228684 |
| E_g_godlewskii_SY11 | MG194573 | MG228495 | MG228685 |
| E_g_godlewskii_SY12 | MG194574 | MG228496 | MG228686 |
| E_g_godlewskii_SY13 | MG194575 | MG228497 | MG228687 |
| E_g_godlewskii_SY2 | MG194576 | MG228498 | MG228688 |
| E_g_godlewskii_SY3 | MG194577 | MG228499 | MG228689 |
| E_g_godlewskii_SY4 | MG194578 | MG228500 | MG228690 |
| E_g_godlewskii_SY5 | MG194579 | MG228501 | MG228691 |
| E_g_godlewskii_SY6 | MG194580 | MG228502 | MG228692 |
| E_g_godlewskii_SY7 | MG194581 | MG228503 | MG228693 |
| E_g_godlewskii_SY8 | MG194582 | MG228504 | MG228694 |
| E_g_godlewskii_SY9 | MG194583 | MG228505 | MG228695 |
| E_g_godlewskii_XYH10 | MG194584 | MG228506 | MG228696 |
| E_g_godlewskii_XYH11 | MG194585 | MG228507 | MG228697 |
| E_g_godlewskii_XYH2 | MG194586 | MG228508 | MG228698 |
| E_g_godlewskii_XYH3 | MG194587 | MG228509 | MG228699 |
| E_g_godlewskii_XYH4 | MG194588 | MG228510 | MG228700 |
| E_g_godlewskii_XYH5 | MG194589 | MG228511 | MG228701 |
| E_g_godlewskii_XYH6 | MG194590 | MG228512 | MG228702 |
| E_g_godlewskii_XYH7 | MG194591 | MG228513 | MG228703 |
| E_g_godlewskii_XYH8 | MG194592 | MG228514 | MG228704 |
| E_g_godlewskii_XYH9 | MG194593 | MG228515 | MG228705 |
| E_g_khamensis_BS1 | MG194594 | MG228516 | MG228706 |
| E_g_khamensis_BS3 | MG194595 | MG228517 | MG228707 |
| E_g_khamensis_BS4 | MG194596 | MG228518 | MG228708 |
| E_g_khamensis_BS5 | MG194597 | MG228519 | MG228709 |
| E_g_khamensis_CD1 | MG194598 | MG228520 | MG228710 |
| E_g_khamensis_LS1 | MG194599 | MG228521 | MG228711 |
| E_g_khamensis_LS2 | MG194600 | MG228522 | MG228712 |
| E_g_khamensis_LS3 | MG194601 | MG228523 | MG228713 |
| E_g_khamensis_LS4 | MG194602 | MG228524 | MG228714 |
| E_g_khamensis_LZ1 | MG194603 | MG228525 | MG228715 |
| E_g_khamensis_LZ2 | MG194604 | MG228526 | MG228716 |
| E_g_khamensis_LZ3 | MG194605 | MG228527 | MG228717 |
| E_g_khamensis_LZ4 | MG194606 | MG228528 | MG228718 |
| E_g_khamensis_LZ5 | MG194607 | MG228529 | MG228719 |
| E_g_khamensis_LZ6 | MG194608 | MG228530 | MG228720 |
| E_g_khamensis_LZ7 | MG194609 | MG228531 | MG228721 |
| E_g_khamensis_LZ8 | MG194610 | MG228532 | MG228722 |
| E_g_khamensis_MK1 | MG194611 | MG228533 | MG228723 |
| E_g_khamensis_MK2 | MG194612 | MG228534 | MG228724 |
| E_g_khamensis_XS1 | MG194613 | MG228535 | MG228725 |
| E_g_khamensis_XS2 | MG194614 | MG228536 | MG228726 |
| E_g_khamensis_XS4 | MG194615 | MG228537 | MG228727 |
| E_g_khamensis_XS5 | MG194616 | MG228538 | MG228728 |
| E_g_omissa_BJ1 | MG194617 | MG228539 | MG228729 |
| E_g_omissa_BJ2 | MG194618 | MG228540 | MG228730 |
| E_g_omissa_HB1 | MG194619 | MG228541 | MG228731 |
| E_g_omissa_HB10 | MG194620 | MG228542 | MG228732 |
| E_g_omissa_HB11 | MG194621 | MG228543 | MG228733 |
| E_g_omissa_HB12 | MG194622 | MG228544 | MG228734 |
| E_g_omissa_HB13 | MG194623 | MG228545 | MG228735 |
| E_g_omissa_HB2 | MG194624 | MG228546 | MG228736 |
| E_g_omissa_HB3 | MG194625 | MG228547 | MG228737 |
| E_g_omissa_HB4 | MG194626 | MG228548 | MG228738 |
| E_g_omissa_HB5 | MG194627 | MG228549 | MG228739 |
| E_g_omissa_HB6 | MG194628 | MG228550 | MG228740 |
| E_g_omissa_HB7 | MG194629 | MG228551 | MG228741 |
| E_g_omissa_HB8 | MG194630 | MG228552 | MG228742 |
| E_g_omissa_HB9 | MG194631 | MG228553 | MG228743 |
| E_g_omissa_JY1 | MG194632 | MG228554 | MG228744 |
| E_g_omissa_JY2 | MG194633 | MG228555 | MG228745 |
| E_g_omissa_JY4 | MG194634 | MG228556 | MG228746 |
| E_g_omissa_JY6 | MG194635 | MG228557 | MG228747 |
| E_g_omissa_JY7 | MG194636 | MG228558 | MG228748 |
| E_g_omissa_JY8 | MG194637 | MG228559 | MG228749 |
| E_g_omissa_PL1 | MG194638 | MG228560 | MG228750 |
| E_g_omissa_PL2 | MG194639 | MG228561 | MG228751 |
| E_g_omissa_SX1 | MG194640 | MG228562 | MG228752 |
| E_g_omissa_SX10 | MG194641 | MG228563 | MG228753 |
| E_g_omissa_SX11 | MG194642 | MG228564 | MG228754 |
| E_g_omissa_SX12 | MG194643 | MG228565 | MG228755 |
| E_g_omissa_SX13 | MG194644 | MG228566 | MG228756 |
| E_g_omissa_SX14 | MG194645 | MG228567 | MG228757 |
| E_g_omissa_SX15 | MG194646 | MG228568 | MG228758 |
| E_g_omissa_SX16 | MG194647 | MG228569 | MG228759 |
| E_g_omissa_SX17 | MG194648 | MG228570 | MG228760 |
| E_g_omissa_SX18 | MG194649 | MG228571 | MG228761 |
| E_g_omissa_SX19 | MG194650 | MG228572 | MG228762 |
| E_g_omissa_SX2 | MG194651 | MG228573 | MG228763 |
| E_g_omissa_SX20 | MG194652 | MG228574 | MG228764 |
| E_g_omissa_SX21 | MG194653 | MG228575 | MG228765 |
| E_g_omissa_SX22 | MG194654 | MG228576 | MG228766 |
| E_g_omissa_SX23 | MG194655 | MG228577 | MG228767 |
| E_g_omissa_SX24 | MG194656 | MG228578 | MG228768 |
| E_g_omissa_SX25 | MG194657 | MG228579 | MG228769 |
| E_g_omissa_SX26 | MG194658 | MG228580 | MG228770 |
| E_g_omissa_SX3 | MG194659 | MG228581 | MG228771 |
| E_g_omissa_SX4 | MG194660 | MG228582 | MG228772 |
| E_g_omissa_SX5 | MG194661 | MG228583 | MG228773 |
| E_g_omissa_SX6 | MG194662 | MG228584 | MG228774 |
| E_g_omissa_SX7 | MG194663 | MG228585 | MG228775 |
| E_g_omissa_SX8 | MG194664 | MG228586 | MG228776 |
| E_g_omissa_SX9 | MG194665 | MG228587 | MG228777 |
| E_g_styani_CZS1 | MG194666 | MG228588 | MG228778 |
| E_g_styani_SP1 | MG194667 | MG228589 | MG228779 |
| E_g_styani_SP10 | MG194668 | MG228590 | MG228780 |
| E_g_styani_SP11 | MG194669 | MG228591 | MG228781 |
| E_g_styani_SP12 | MG194670 | MG228592 | MG228782 |
| E_g_styani_SP13 | MG194671 | MG228593 | MG228783 |
| E_g_styani_SP14 | MG194672 | MG228594 | MG228784 |
| E_g_styani_SP15 | MG194673 | MG228595 | MG228785 |
| E_g_styani_SP16 | MG194674 | MG228596 | MG228786 |
| E_g_styani_SP17 | MG194675 | MG228597 | MG228787 |
| E_g_styani_SP18 | MG194676 | MG228598 | MG228788 |
| E_g_styani_SP2 | MG194677 | MG228599 | MG228789 |
| E_g_styani_SP4 | MG194678 | MG228600 | MG228790 |
| E_g_styani_SP6 | MG194679 | MG228601 | MG228791 |
| E_g_styani_SP7 | MG194680 | MG228602 | MG228792 |
| E_g_styani_SP8 | MG194681 | MG228603 | MG228793 |
| E_g_styani_SP9 | MG194682 | MG228604 | MG228794 |
| E_g_yunnanensis_DQ2 | MG194683 | MG228605 | MG228795 |
| E_g_yunnanensis_DR1 | MG194684 | MG228606 | MG228796 |
| E_g_yunnanensis_DR2 | MG194685 | MG228607 | MG228797 |
| E_g_yunnanensis_GZ1 | MG194686 | MG228608 | MG228798 |
| E_g_yunnanensis_GZ2 | MG194687 | MG228609 | MG228799 |
| E_g_yunnanensis_GZ3 | MG194688 | MG228610 | MG228800 |
| E_g_yunnanensis_JZS10 | MG194689 | MG228611 | MG228801 |
| E_g_yunnanensis_JZS11 | MG194690 | MG228612 | MG228802 |
| E_g_yunnanensis_JZS12 | MG194691 | MG228613 | MG228803 |
| E_g_yunnanensis_JZS13 | MG194692 | MG228614 | MG228804 |
| E_g_yunnanensis_JZS14 | MG194693 | MG228615 | MG228805 |
| E_g_yunnanensis_JZS15 | MG194694 | MG228616 | MG228806 |
| E_g_yunnanensis_JZS2 | MG194695 | MG228617 | MG228807 |
| E_g_yunnanensis_JZS3 | MG194696 | MG228618 | MG228808 |
| E_g_yunnanensis_JZS4 | MG194697 | MG228619 | MG228809 |
| E_g_yunnanensis_JZS6 | MG194698 | MG228620 | MG228810 |
| E_g_yunnanensis_JZS7 | MG194699 | MG228621 | MG228811 |
| E_g_yunnanensis_KM1 | MG194700 | MG228622 | MG228812 |
| E_g_yunnanensis_KM10 | MG194701 | MG228623 | MG228813 |
| E_g_yunnanensis_KM11 | MG194702 | MG228624 | MG228814 |
| E_g_yunnanensis_KM12 | MG194703 | MG228625 | MG228815 |
| E_g_yunnanensis_KM13 | MG194704 | MG228626 | MG228816 |
| E_g_yunnanensis_KM2 | MG194705 | MG228627 | MG228817 |
| E_g_yunnanensis_KM3 | MG194706 | MG228628 | MG228818 |
| E_g_yunnanensis_KM4 | MG194707 | MG228629 | MG228819 |
| E_g_yunnanensis_KM5 | MG194708 | MG228630 | MG228820 |
| E_g_yunnanensis_KM6 | MG194709 | MG228631 | MG228821 |
| E_g_yunnanensis_KM7 | MG194710 | MG228632 | MG228822 |
| E_g_yunnanensis_KM8 | MG194711 | MG228633 | MG228823 |
| E_g_yunnanensis_KM9 | MG194712 | MG228634 | MG228824 |
| E_g_yunnanensis_LJ1 | MG194713 | MG228635 | MG228825 |
| E_g_yunnanensis_LJ10 | MG194714 | MG228636 | MG228826 |
| E_g_yunnanensis_LJ11 | MG194715 | MG228637 | MG228827 |
| E_g_yunnanensis_LJ12 | MG194716 | MG228638 | MG228828 |
| E_g_yunnanensis_LJ13 | MG194717 | MG228639 | MG228829 |
| E_g_yunnanensis_LJ2 | MG194718 | MG228640 | MG228830 |
| E_g_yunnanensis_LJ3 | MG194719 | MG228641 | MG228831 |
| E_g_yunnanensis_LJ4 | MG194720 | MG228642 | MG228832 |
| E_g_yunnanensis_LJ5 | MG194721 | MG228643 | MG228833 |
| E_g_yunnanensis_LJ6 | MG194722 | MG228644 | MG228834 |
| E_g_yunnanensis_LJ7 | MG194723 | MG228645 | MG228835 |
| E_g_yunnanensis_LJ8 | MG194724 | MG228646 | MG228836 |
| E_g_yunnanensis_LJ9 | MG194725 | MG228647 | MG228837 |
| E_g_yunnanensis_MLS1 | MG194726 | MG228648 | MG228838 |
| E_g_yunnanensis_MLS2 | MG194727 | MG228649 | MG228839 |
| E_g_yunnanensis_MLS3 | MG194728 | MG228650 | MG228840 |

**Table S5**. Genbank Accession numbers for individuals of nuclear genes used in this study.

| Individual ID | Genbank Accession Number | |
| --- | --- | --- |
|  | *FIB* | *MUSK* |
| E_c_decolorata_HJ1 | MG228841 | MG228946 |
| E_c_decolorata_HJ2 | MG228842 | MG228947 |
| E_c_decolorata_HJ3 | MG228843 | MG228948 |
| E_c_decolorata_HJ4 | MG228844 | MG228949 |
| E_c_decolorata_HJ5 | MG228845 | MG228950 |
| E_c_decolorata_HJ6 | MG228846 | MG228951 |
| E_c_decolorata_HJ7 | MG228847 | MG228952 |
| E_c_decolorata_UR1 | MG228848 | MG228953 |
| E_c_decolorata_UR2 | MG228849 | MG228954 |
| E_c_decolorata_UR3 | MG228850 | MG228955 |
| E_c_godlewskii_DDS1 | MG228851 | MG228956 |
| E_c_godlewskii_DDS10 | MG228852 | MG228957 |
| E_c_godlewskii_DDS11 | MG228853 | MG228958 |
| E_c_godlewskii_DDS3 | MG228854 | MG228959 |
| E_c_godlewskii_DDS6 | MG228855 | MG228960 |
| E_c_godlewskii_DDS8 | MG228856 | MG228961 |
| E_c_godlewskii_DDS9 | MG228857 | MG228962 |
| E_c_godlewskii_LAN1 | MG228858 | MG228963 |
| E_c_godlewskii_SY1 | MG228859 | MG228964 |
| E_c_godlewskii_SY11 | MG228860 | MG228965 |
| E_c_godlewskii_SY2 | MG228861 | MG228966 |
| E_c_godlewskii_SY3 | MG228862 | MG228967 |
| E_c_godlewskii_SY4 | MG228863 | MG228968 |
| E_c_godlewskii_SY5 | MG228864 | MG228969 |
| E_c_godlewskii_SY8 | MG228865 | MG228970 |
| E_c_godlewskii_SY9 | MG228866 | MG228971 |
| E_c_godlewskii_XYH1 | MG228867 | MG228972 |
| E_c_godlewskii_XYH2 | MG228868 | MG228973 |
| E_c_godlewskii_XYH4 | MG228869 | MG228974 |
| E_c_godlewskii_XYH5 | MG228870 | MG228975 |
| E_c_godlewskii_XYH7 | MG228871 | MG228976 |
| E_c_godlewskii_XYH9 | MG228872 | MG228977 |
| E_c_khamensis_LS1 | MG228873 | MG228978 |
| E_c_khamensis_LS2 | MG228874 | MG228979 |
| E_c_khamensis_LS3 | MG228875 | MG228980 |
| E_c_khamensis_LZ1 | MG228876 | MG228981 |
| E_c_khamensis_LZ2 | MG228877 | MG228982 |
| E_c_khamensis_LZ3 | MG228878 | MG228983 |
| E_c_khamensis_LZ4 | MG228879 | MG228984 |
| E_c_khamensis_LZ5 | MG228880 | MG228985 |
| E_c_khamensis_LZ6 | MG228881 | MG228986 |
| E_c_khamensis_LZ7 | MG228882 | MG228987 |
| E_c_khamensis_LZ8 | MG228883 | MG228988 |
| E_c_khamensis_MK1 | MG228884 | MG228989 |
| E_c_omissa_BJ1 | MG228885 | MG228990 |
| E_c_omissa_HB1 | MG228886 | MG228991 |
| E_c_omissa_HB2 | MG228887 | MG228992 |
| E_c_omissa_HB4 | MG228888 | MG228993 |
| E_c_omissa_HB5 | MG228889 | MG228994 |
| E_c_omissa_HB6 | MG228890 | MG228995 |
| E_c_omissa_JY1 | MG228891 | MG228996 |
| E_c_omissa_JY3 | MG228892 | MG228997 |
| E_c_omissa_JY4 | MG228893 | MG228998 |
| E_c_omissa_JY6 | MG228894 | MG228999 |
| E_c_omissa_JY7 | MG228895 | MG229000 |
| E_c_omissa_PL1 | MG228896 | MG229001 |
| E_c_omissa_PL2 | MG228897 | MG229002 |
| E_c_omissa_SX10 | MG228898 | MG229003 |
| E_c_omissa_SX12 | MG228899 | MG229004 |
| E_c_omissa_SX16 | MG228900 | MG229005 |
| E_c_omissa_SX17 | MG228901 | MG229006 |
| E_c_omissa_SX18 | MG228902 | MG229007 |
| E_c_omissa_SX19 | MG228903 | MG229008 |
| E_c_omissa_SX21 | MG228904 | MG229009 |
| E_c_omissa_SX24 | MG228905 | MG229010 |
| E_c_omissa_SX26 | MG228906 | MG229011 |
| E_c_omissa_SX3 | MG228907 | MG229012 |
| E_c_omissa_SX4 | MG228908 | MG229013 |
| E_c_omissa_SX5 | MG228909 | MG229014 |
| E_c_omissa_SX7 | MG228910 | MG229015 |
| E_c_omissa_SX8 | MG228911 | MG229016 |
| E_c_styani_SP1 | MG228912 | MG229017 |
| E_c_styani_SP12 | MG228913 | MG229018 |
| E_c_styani_SP13 | MG228914 | MG229019 |
| E_c_styani_SP2 | MG228915 | MG229020 |
| E_c_styani_SP4 | MG228916 | MG229021 |
| E_c_styani_SP5 | MG228917 | MG229022 |
| E_c_styani_SP8 | MG228918 | MG229023 |
| E_c_styani_SP9 | MG228919 | MG229024 |
| E_c_yunnanensis_GZ1 | MG228920 | MG229025 |
| E_c_yunnanensis_JZS10 | MG228921 | MG229026 |
| E_c_yunnanensis_JZS11 | MG228922 | MG229027 |
| E_c_yunnanensis_JZS12 | MG228923 | MG229028 |
| E_c_yunnanensis_JZS14 | MG228924 | MG229029 |
| E_c_yunnanensis_JZS15 | MG228925 | MG229030 |
| E_c_yunnanensis_JZS3 | MG228926 | MG229031 |
| E_c_yunnanensis_JZS4 | MG228927 | MG229032 |
| E_c_yunnanensis_JZS6 | MG228928 | MG229033 |
| E_c_yunnanensis_JZS7 | MG228929 | MG229034 |
| E_c_yunnanensis_JZS9 | MG228930 | MG229035 |
| E_c_yunnanensis_KM10 | MG228931 | MG229036 |
| E_c_yunnanensis_KM11 | MG228932 | MG229037 |
| E_c_yunnanensis_KM12 | MG228933 | MG229038 |
| E_c_yunnanensis_KM13 | MG228934 | MG229039 |
| E_c_yunnanensis_KM2 | MG228935 | MG229040 |
| E_c_yunnanensis_KM4 | MG228936 | MG229041 |
| E_c_yunnanensis_KM5 | MG228937 | MG229042 |
| E_c_yunnanensis_KM7 | MG228938 | MG229043 |
| E_c_yunnanensis_MLS1 | MG228939 | MG229044 |
| E_c_yunnanensis_MLS4 | MG228940 | MG229045 |
| E_c_yunnanensis_LJ9 | MG228941 | MG229046 |
| E_c_yunnanensis_LJ10 | MG228942 | MG229047 |
| E_c_yunnanensis_LJ11 | MG228943 | MG229048 |
| E_c_yunnanensis_LJ12 | MG228944 | MG229049 |
| E_c_yunnanensis_LJ13 | MG228945 | MG229050 |

**Table S6**. Estimations of posterior distributions of parameters revealed by DIY-ABC for the best scenario of demographic history of northern and southern population, respectively.

|  | Parameter | N1 | t1 | Na |
| --- | --- | --- | --- | --- |
|  | Mean | 3.18e+006 | 3.92e+004 | 1.47e+005 |
| Northern lineage | Median | 3.09e+006 | 3.10e+004 | 1.30e+005 |
|  | Mode | 2.48e+006 | 1.19e+004 | 9.18e+004 |
|  | 95% CI | 7.94e+005 5.74e+006 | 6.76e+003 1.00e+005 | 4.09e+004 3.05e+005 |
|  | Mean | 2.42e+006 | 4.94e+004 | 1.16e+005 |
| Southern lineage | Median | 2.27e+006 | 3.86e+004 | 1.08e+005 |
|  | Mode | 1.74e+006 | 1.41e+004 | 6.58e+004 |
|  | 95% CI | 5.89e+005 4.64e+006 | 9.22e+003 1.28e+005 | 3.25e+004 2.24e+005 |

Estimation was based on 1% of the closest simulated data sets and logistic transformation of parameters. Descriptions of all used parameters were listed in Table S3.


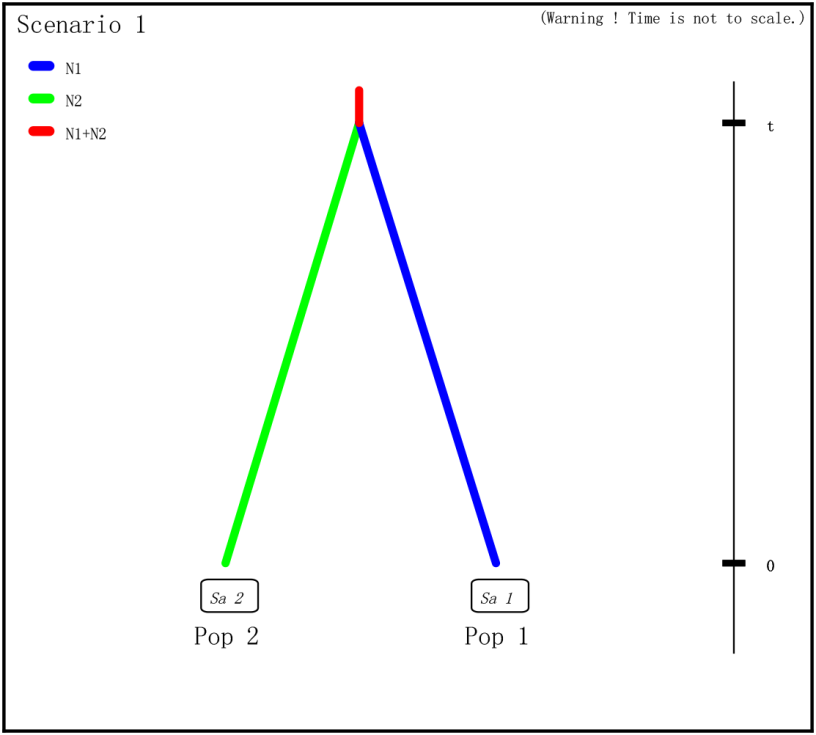


Fig. S1 Demographic expansion and divergence models of *Emberiza godlewskii*. ABC results showing demographic history. Effective population sizes are marked in different colours and times of events (not to scale) are indicated.


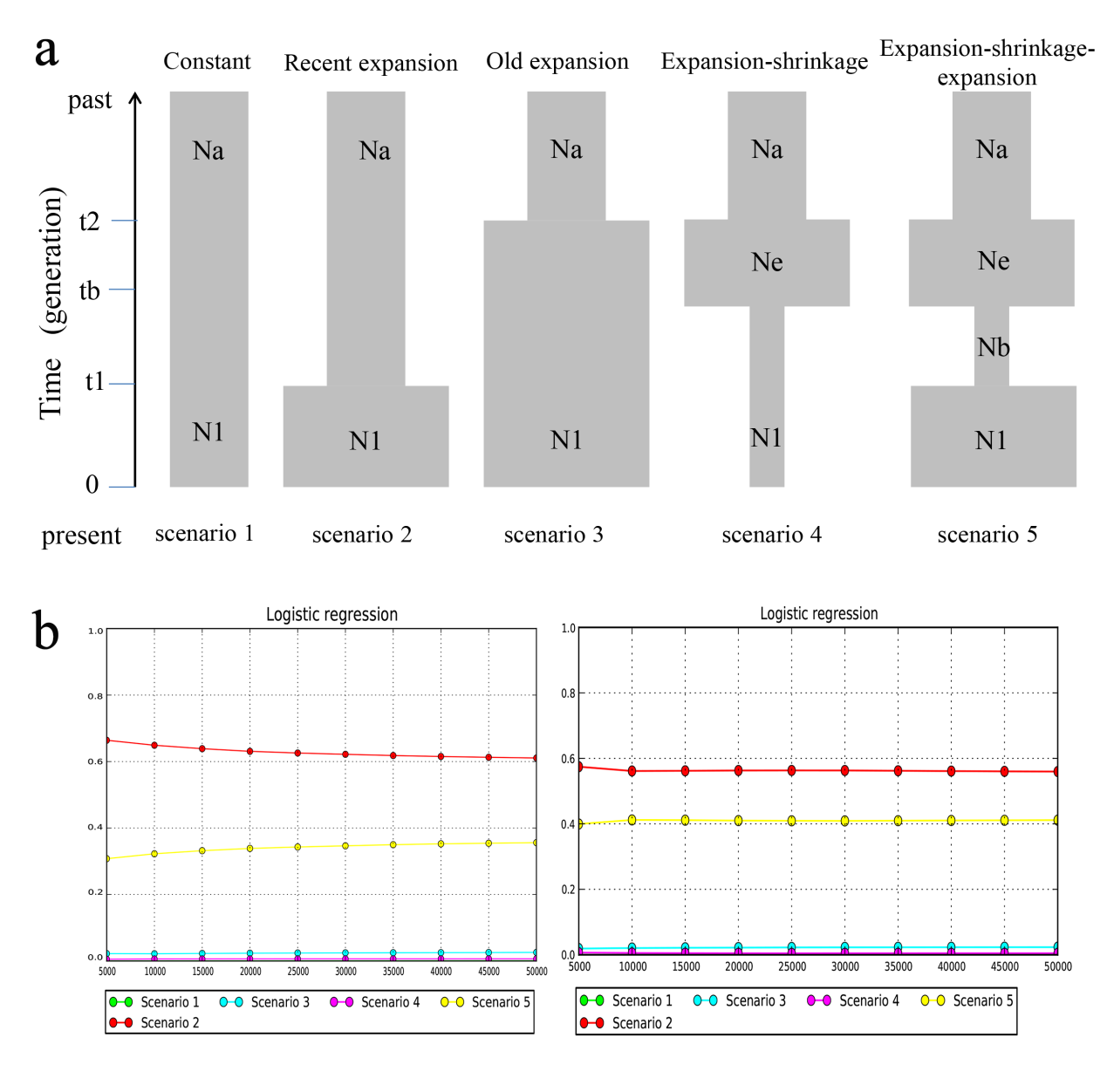
 Fig. S2 (a) Schematic representation of ABC modelling of changes in population sizes. Na, ancestral population size; N1, current population size; Ne and Nb, population sizes between Na and N1; t2, old expansion time; tb, bottleneck time; t1, recent expansion time. (b) Posterior probabilities obtained by logistic regression of 1% of the closest simulated datasets for the northern and southern lineages, respectively, from left to right.
